# Supplementary figures and images for: Comparative analysis of multiorgan toxicity induced by long term use of disease modifying anti-rheumatic drugs
Source: PLoS One. 2023 Aug 25;18(8):e0290668. doi: 10.1371/journal.pone.0290668 (PMC10456141; doi:10.1371/journal.pone.0290668)

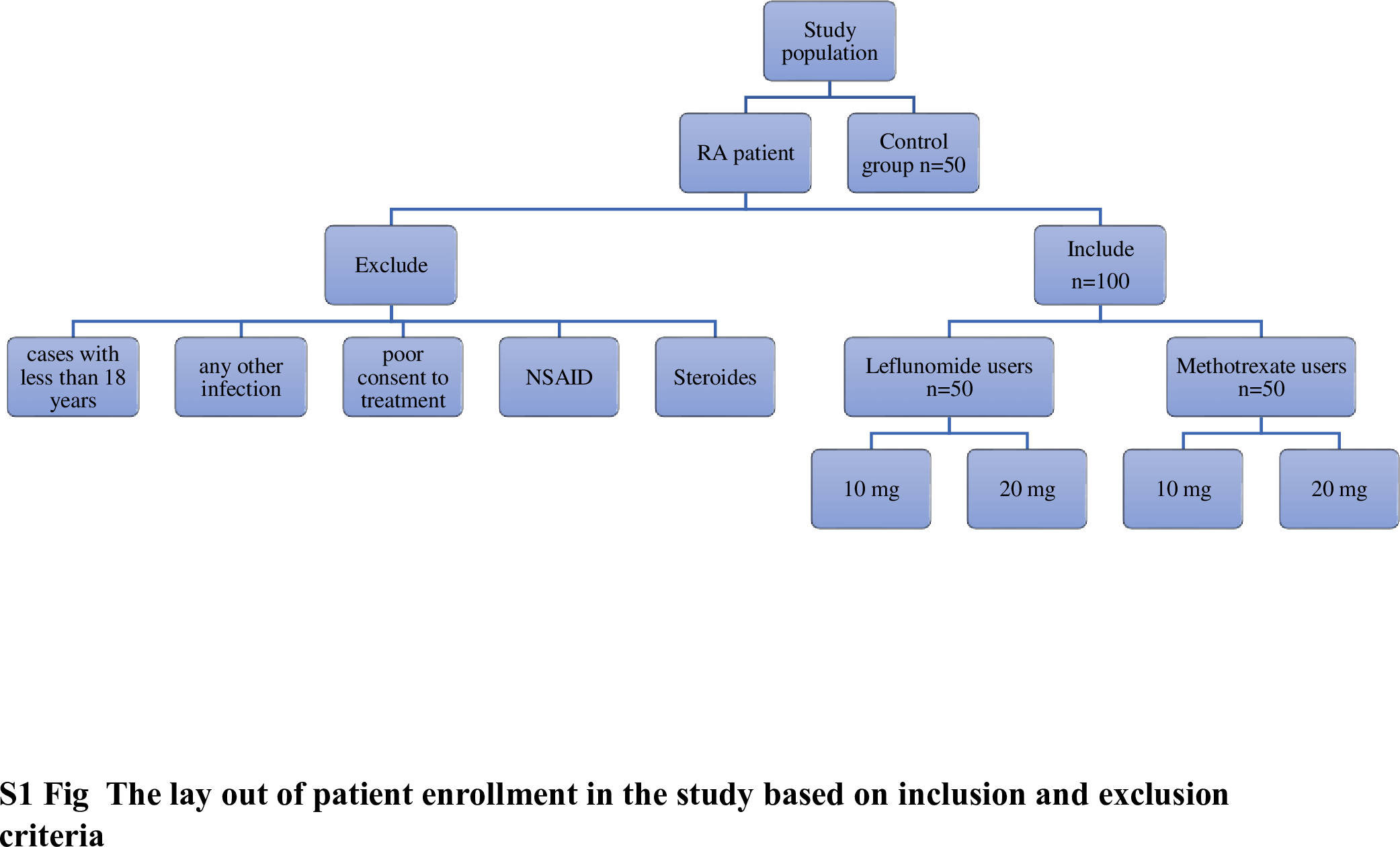

Supplement: S1 Fig — (TIF) [file pone.0290668.s001.tif]
