# Supplementary material for: Comparative analysis of multiorgan toxicity induced by long term use of disease modifying anti-rheumatic drugs
Source: PLoS One. 2023 Aug 25;18(8):e0290668. doi: 10.1371/journal.pone.0290668 (PMC10456141; doi:10.1371/journal.pone.0290668)
Supplement: S1 File — (PDF) [file pone.0290668.s002.pdf]

Rheumatoid Arthritis Questionnaire

Consent: I hereby willingly and voluntarily participate in the study. All details about study have been explained to me and I would contribute by providing 3cc blood sample to check the effect of treatment I received. All the disease and demographic information will be kept confidential and used only for purposed research plan.

Signature ____________ Date ________________

Patient ID ___________ Gender: Female ☐ Male ☐

Marital status: Married Single Widow/widower No. of kids: _________

Occupation: _________________________ Socio-economic status: _______________________

Home Address ____

Contact No __________________________

Addictions:

Caffeine/Nicotine/Cigarette/Hukkah/Naswar/Alcohol/Anti-depressants/Betel leaf/others

Eating Routine and Frequency

Tea/Coffee/Milk/soft Drinks/Gluten/Red meat/fried food/pizza/ burger/Pastries/ Fermented milk/Yogurt/Cream/Butter /Cheese/Casein/ Custard/Ice cream/Brinjal/ Peppers/ Tomatoes/ Potatoes____

Relief Pain by using

Heat therapy/Exercise______________________

Onion/cucumber/sweet potato/Yogurt/Aloe Vera/Ginger/Turmeric/Peas and beans/Nuts/Fish/ Chicken____

Vital Signs

Blood Pressure Temperature Blood Sugar

Height (m) Weight (kg) BMI

Medicines Used: DMARDS,

☐ Leflunomide ☐ Methotrexate ☐ Sulfasalazine ☐ Hydrxychloroquine

☐ Meloxicam ☐ Prednisolone ☐ Celecoxib

Steroids

NASIDs

Medication for other illness

____

____

Duration of medicine ______________________ Dose of medicine _______________________

Adverse effect of medicine

Nausea/Headache/Dizziness /Rash/Fatigue /Cough/Diarrhea /Alopecia/Stomatitis/Abdominal pain

Details of reported adverse effects (if any)

Any other information
