# Supplementary material for: Comparative analysis of multiorgan toxicity induced by long term use of disease modifying anti-rheumatic drugs
Source: PLoS One. 2023 Aug 25;18(8):e0290668. doi: 10.1371/journal.pone.0290668 (PMC10456141; doi:10.1371/journal.pone.0290668)
Supplement: S1 Table — (PDF) [file pone.0290668.s003.pdf]

S1 Table Minimal Data set of healthy control group

ID	Age	Wt (kg)	Ht (m)	BMI (kg/m^2)	SBP (mm Hg)	DBP (mm Hg)	SPO$_2$ (%)	Pulse Rate (BPM)	Body Temp. (◦C)	ALP (U/L)	ALT (U/L)	AST (U/L)	T bili (mg/dl)	D bili (mg/dl)	In bili (mg/dl)	SCr	GFR	BUN	Urinary Urea	Uric Acid
C1	41	57	1.64	21.2	110	70	96	84	37	202	10	24	1	0.6	0.4	0.8	102.3	18	13	4.5
C2	32	67	1.69	23.5	100	90	94	99	37	240	12	14	0.9	0.4	0.5	0.9	89.7	19	14	8
C3	41	53	1.61	20.4	110	70	98	78	37	300	15	28	0.9	0.4	0.5	0.8	102.3	18	20	7.2
C4	36	57.3	1.52	24.8	120	80	97	98	37	295	14	23	0.9	0.6	0.3	0.7	120.2	15	15	7.9
C5	39	46	1.56	18.9	110	70	98	65	37	284	16	27	0.7	0.4	0.3	0.8	103.2	22	17	5.2
C6	42	54	1.54	22.7	100	70	99	74	37	301	17	28	0.6	0.4	0.2	0.9	91.3	20	19	6.1
C7	45	64	1.61	24.7	130	80	96	84	37	300	13	25	0.9	0.5	0.4	0.7	119.4	13	16	6.3
C8	65	50	1.56	20.5	110	70	94	89	37	306	25	16	0.9	0.6	0.3	0.9	89.7	17	14	5.9
C9	25	60	1.56	24.6	120	80	95	90	37	277	21	24	1.1	0.7	0.5	1	79.1	14	16	5
C10	35	56	1.66	20.3	110	70	96	91	37	191	24	32	0.6	0.4	0.2	0.8	101.9	19	15	7.9
C11	32	57	1.64	21.2	110	70	96	92	37	245	17	30	1	0.6	0.4	0.8	101.1	22	17	5.3
C12	55	62	1.59	24.8	120	80	94	94	37	254	15	25	0.9	0.4	0.5	0.7	118	18	16	5
C13	42	65	1.64	24.2	110	70	98	96	37	230	13	30	0.7	0.2	0.5	0.8	100.4	21	14	6.7
C14	18	52	1.62	19.8	110	70	98	99	37	255	26	31	0.9	0.6	0.3	0.7	117.1	18	17	4.9
C15	40	53	1.62	20.2	110	70	98	76	37	267	17	23	0.7	0.4	0.3	0.8	100.7	23	18	5.1
C16	34	53	1.59	21	110	70	99	75	37	290	14	22	0.6	0.4	0.2	0.8	101.5	18	15	8.2
C17	51	52	1.54	21.9	110	70	96	74	37	230	23	25	0.9	0.5	0.4	0.9	89.7	19	15	6.7
C18	31	53	1.54	22.3	110	70	97	89	37	222	12	18	0.9	0.6	0.3	0.8	79.02	22	17	7.9
C19	39	48	1.56	19.7	120	80	95	87	37	234	20	55	1.2	0.7	0.5	0.9	80.2	24	18	8
C20	31	50	1.61	19.3	110	70	96	93	37	282	24	37	0.6	0.4	0.2	0.8	103.6	22	14	5.3
C21	51	64	1.61	24.7	130	80	96	84	37	301	13	25	0.9	0.5	0.4	0.8	104.5	23	14	6.7
C22	39	50	1.56	20.5	110	70	94	89	37	306	25	16	0.9	0.6	0.3	0.8	102.3	18	17	4.9
C23	45	60	1.56	24.6	120	80	95	90	37	277	21	25	1.1	0.7	0.5	0.9	89.7	19	18	5.1
C24	29	56	1.66	20.3	110	70	96	91	37	191	24	31	0.6	0.4	0.2	0.8	102.3	18	15	8.2
C25	32	57	1.64	21.2	110	70	96	84	37	202	10	24	1	0.6	0.4	0.7	120.2	15	15	6.7

C26	45	67	1.69	23.5	100	90	94	99	37	240	12	14	0.9	0.4	0.5	0.8	103.2	22	17	7.9
C27	36	54	1.54	22.7	100	70	99	74	37	299	17	29	0.6	0.4	0.2	0.9	91.3	20	18	8
C28	39	64	1.61	24.7	130	80	96	84	37	300	13	25	0.9	0.5	0.4	0.7	119.4	13	13	4.5
C29	42	56	1.66	20.3	110	70	96	91	37	191	24	31	0.6	0.4	0.2	0.9	89.7	17	14	8
C30	45	57	1.64	21.2	110	70	96	92	37	245	17	30	1	0.6	0.4	0.8	84.2	16	20	7.2
C31	49	52	1.54	21.9	110	70	96	74	37	230	23	25	0.9	0.5	0.4	0.8	101.9	19	15	7.9
C32	48	53	1.54	22.3	110	70	97	89	37	222	12	18	0.9	0.6	0.3	0.8	101.1	22	14	6.7
C33	47	53	1.59	21	110	70	99	75	37	290	14	22	0.6	0.4	0.2	0.7	118	18	17	4.9
C34	51	52	1.54	21.9	110	70	96	74	37	230	23	25	0.9	0.5	0.4	0.8	100.4	21	18	5.1
C35	33	53	1.54	22.3	110	70	97	89	37	222	12	18	0.9	0.6	0.3	0.7	117.1	18	15	8.2
C36	39	48	1.56	19.7	120	80	95	87	37	234	20	55	1.2	0.7	0.5	0.8	100.7	23	15	6.7
C37	37	50	1.61	19.3	110	70	96	93	37	282	24	37	0.6	0.4	0.2	0.8	101.5	18	17	7.9
C38	39	64	1.61	24.7	130	80	96	84	37	300	13	25	0.9	0.5	0.4	0.9	89.7	19	18	8
C39	46	50	1.56	20.5	110	70	94	89	37	306	25	16	0.9	0.6	0.3	0.8	79.08	22	14	5.3
C40	41	60	1.56	24.6	120	80	95	90	37	277	21	24	1.1	0.7	0.5	0.9	84.2	21	14	8
C41	34	56	1.66	20.3	110	70	96	91	37	191	24	32	0.6	0.4	0.2	0.8	103.6	22	20	7.2
C42	59	57	1.64	21.2	110	70	96	84	37	202	10	24	1	0.6	0.4	0.8	104.5	23	15	7.9
C43	46	46	1.56	18.9	110	70	98	65	37	284	16	27	0.7	0.4	0.3	0.9	89.7	17	17	5.2
C44	44	54	1.54	22.7	100	70	99	74	37	299	17	29	0.6	0.4	0.2	0.9	78.9	15	19	6.1
C45	35	63.5	1.61	24.7	130	80	96	84	37	289	13	25	0.9	0.5	0.4	0.8	101.9	19	16	6.3
C46	39	50	1.57	20.5	110	70	94	89	37	306	25	16	0.9	0.6	0.3	0.8	101.1	22	14	5.9
C47	41	46	1.56	18.9	110	70	98	65	37	284	16	27	0.7	0.4	0.3	0.7	118.1	18	16	5
C48	40	54	1.54	22.7	100	70	99	74	37	298	17	29	0.6	0.4	0.2	0.7	120.4	13	15	7.9
C49	38	64	1.61	24.7	130	80	96	84	37	302	13	25	0.9	0.5	0.4	0.9	110.4	17	17	5.3
C50	47	50	1.56	20.5	110	70	94	89	37	306	25	16	0.9	0.6	0.3	0.9	78.4	13	16	5
