# Supplementary material for: Comparative analysis of multiorgan toxicity induced by long term use of disease modifying anti-rheumatic drugs
Source: PLoS One. 2023 Aug 25;18(8):e0290668. doi: 10.1371/journal.pone.0290668 (PMC10456141; doi:10.1371/journal.pone.0290668)
Supplement: S2 Table — (PDF) [file pone.0290668.s004.pdf]

S2 Table Minimal Data set of RA patients treated with Methotrexate

ID	Age (yr)	Wt (kg)	Ht (m)	BMI (kg/m^2)	SBP (mm Hg)	DBP (mm Hg)	SPO_2 (%)	Pulse Rate (BPM)	Body Temp. (◦C)	ALP (U/L)	ALT (U/L)	AST (U/L)	T bili (mg/dl)	D bili (mg/dl)	In bili (mg/d	SCr	GFR	BUN	Urea	Uric Acid
RM1	41	63	1.61	24.3	130	90	96	102	37	286	51	45	0.8	0.4	0.4	2.7	18.9	18	5.1	2.9
RM2	32	59	1.47	27.3	120	60	95	94	37	301	57	54	1	0.5	0.5	1.2	47	106	4.9	2.7
RM3	41	59	1.5	26.2	130	80	96	108	37	256	49	44	0.7	0.4	0.3	0.6	110.7	20	3.7	2
RM4	35	96	1.42	47.7	130	80	95	115	39	300	59	56	1	0.5	0.5	0.8	76.2	18	5.6	3.8
RM5	65	52	1.5	23.1	140	80	90	83	37	290	50	45	0.8	0.6	0.2	0.7	93.2	31	7.4	3.4
RM6	25	58	1.46	27.2	140	80	98	116	39	260	47	41	0.9	0.6	0.3	0.7	93.6	20	5.7	2.8
RM7	35	63	1.46	29.5	120	80	88	84	37	299	58	51	1	0.4	0.6	2.9	16.4	22	5.9	2.5
RM8	32	71	1.39	36.7	120	80	90	92	37	300	59	54	1	0.5	0.5	0.8	102.3	24	4.8	1.9
RM9	55	53	1.41	26.7	120	80	97	81	37	240	38	35	0.6	0.3	0.3	2.4	27.6	23	6.5	3.1
RM10	42	82	1.46	38.4	140	80	93	122	41	262	44	42	0.8	0.4	0.4	0.8	103.6	21	5.6	2.5
RM11	20	53	1.5	23.5	120	80	88	84	37	298	50	41	0.7	0.3	0.4	0.8	75	20	5.4	2.5
RM12	40	61	1.36	33.1	110	70	94	89	37	280	48	45	0.9	0.5	0.4	1	56.2	32	5.7	3.2
RM13	34	58	1.5	25.7	120	90	83	93	37	305	30	38	0.9	0.4	0.5	4.6	9.7	113	6.4	3.4
RM14	51	60	1.5	26.6	120	80	99	101	39	296	45	42	1	0.6	0.4	0.7	87.9	15	5.6	3.1
RM15	45	70	1.5	31.1	120	80	87	117	41	240	26	38	0.8	0.3	0.5	0.6	99.6	20	5.1	3.3
RM16	38	58	1.5	25.7	120	80	90	83	37	286	51	45	0.8	0.4	0.4	7.4	116.2	16	5.7	2.6
RM17	31	66	1.44	31.8	140	90	98	94	37	562	32	22	1	0.28	0.72	0.8	101.5	27	5.4	3.7
RM18	39	81	1.52	35	120	80	95	115	37	272	21	17	0.62	0.22	0.4	0.7	75.5	22	5.7	3
RM19	39	82	1.49	36.9	140	90	97	89	37	248	18	14	0.5	0.2	0.3	0.8	107.6	22	5.2	2.9
RM20	45	59	1.5	26.2	130	80	96	108	37	256	49	44	0.7	0.4	0.3	1	5.6	81	5.8	2.5
RM21	41	63	1.61	24.3	130	90	96	102	37	286	51	45	0.8	0.4	0.4	4.6	9.7	113	5.1	2.9
RM22	55	59	1.47	27.3	120	60	95	94	37	301	57	54	1	0.5	0.5	0.7	87.9	15	4.9	2.7
RM23	52	59	1.5	26.2	130	80	96	108	37	256	49	44	0.7	0.4	0.3	0.6	99.6	20	3.7	2
RM24	45	96	1.42	47.7	130	80	95	115	39	300	59	56	1	0.5	0.5	7.3	116.9	16	5.6	3.8
RM25	46	53	1.65	23.1	140	80	90	83	37	290	50	45	0.8	0.6	0.2	0.8	101.4	27	7.4	3.4
RM26	43	58	1.46	27.2	140	80	98	116	39	260	47	41	0.9	0.6	0.3	7.4	116.2	16	5.7	2.8

RM27	41	63	1.46	29.5	120	80	88	84	37	299	58	51	0.9	0.4	0.5	2.7	19.8	18	5.9	2.5
RM28	39	71	1.39	36.7	120	80	90	92	37	300	59	54	1	0.5	0.5	1.4	47	106	4.8	1.9
RM29	38	53	1.41	26.7	120	80	97	81	37	240	38	35	0.6	0.3	0.3	0.6	110.7	20	6.5	3.1
RM30	40	72	1.46	33.8	140	80	93	122	41	262	44	42	0.8	0.4	0.4	0.8	76.2	18	5.6	2.5
RM31	32	53	1.5	23.5	120	80	88	84	37	298	50	41	0.7	0.3	0.4	0.7	93.2	31	5.4	2.5
RM32	39	62	1.37	33.1	110	70	94	89	37	280	48	45	0.9	0.5	0.4	0.7	93.6	20	5.7	3.2
RM33	62	58	1.5	25.7	120	90	83	93	37	305	30	38	0.9	0.4	0.5	2.9	16.4	22	6.4	3.4
RM34	53	61	1.51	26.6	120	80	99	101	39	296	45	42	1	0.6	0.4	0.8	102.3	24	5.6	3.1
RM35	61	69	1.52	31.1	120	80	87	117	41	240	26	38	0.8	0.3	0.5	2.4	27.6	23	5.1	3.3
RM36	29	57	1.51	25.7	120	80	90	83	37	286	51	45	0.8	0.4	0.4	0.8	103.6	21	5.7	2.6
RM37	36	67	1.41	31.8	140	90	98	94	37	562	32	22	0.96	0.26	0.7	0.7	19.8	18	5.4	3.7
RM38	38	81	1.52	35	120	80	95	115	37	272	21	17	0.6	0.2	0.4	2.9	47	106	5.7	3
RM39	40	82	1.49	36.9	140	90	97	89	37	248	18	14	0.5	0.2	0.3	0.8	110.7	20	5.2	2.9
RM40	41	59	1.5	26.2	130	80	96	108	37	256	49	44	0.7	0.4	0.3	2.4	76.2	18	5.8	2.5
RM41	45	56	1.42	27.8	130	80	95	115	39	300	59	56	1	0.5	0.5	0.8	93.2	31	5.1	2.9
RM42	42	52	1.5	23.1	140	80	90	83	37	290	50	45	0.8	0.6	0.2	0.8	93.6	20	4.9	2.7
RM43	49	58	1.46	27.2	140	80	98	116	39	260	47	41	0.9	0.6	0.3	1	16.4	22	3.7	2
RM44	59	63	1.46	29.5	120	80	88	84	37	299	58	51	1	0.4	0.6	4.6	102.3	24	5.6	3.8
RM45	52	71	1.39	36.7	120	80	90	92	37	300	59	54	1	0.5	0.5	0.7	27.6	23	7.4	3.4
RM46	36	53	1.41	26.7	120	80	97	81	37	240	38	35	0.6	0.3	0.3	0.6	103.6	21	5.7	2.8
RM47	45	82	1.46	38.4	140	80	93	122	41	262	44	42	0.8	0.4	0.4	7.4	27.6	20	5.9	2.5
RM48	49	53	1.5	23.5	120	80	88	84	37	298	50	41	0.7	0.3	0.4	0.8	103.6	32	4.8	1.9
RM49	42	62	1.36	33.5	110	70	94	89	37	280	48	45	0.9	0.5	0.4	0.7	75	113	5.6	3.1
RM50	48	58	1.5	25.7	120	90	83	93	37	305	30	38	0.9	0.4	0.5	0.8	56.2	15	5.1	3.3

