# Supplementary material for: Comparative analysis of multiorgan toxicity induced by long term use of disease modifying anti-rheumatic drugs
Source: PLoS One. 2023 Aug 25;18(8):e0290668. doi: 10.1371/journal.pone.0290668 (PMC10456141; doi:10.1371/journal.pone.0290668)
Supplement: S3 Table — (PDF) [file pone.0290668.s005.pdf]

S3 Table Minimal Data set of RA patients treated with Leflunomide

ID	Age (yr)	Wt (kg)	Ht (m)	BMI (kg/ m^2)	SBP (mm Hg)	DBP (mm Hg)	SPO_2 (%)	Puls e Rate	Body Temp . (◦C)	ALP (U/L)	AL T (U/L	AST (U/L)	T bili (mg/	D bili (mg/ dl)	In bili (mg/	SCr	GFR	BUN	Urea	Uric Acid
RL1	44	50	1.5	22.2	120	80	94	100	37	289	50	52	0.9	0.5	0.4	0.8	100	26	5.9	2.9
RL2	44	60	1.5	26.6	120	80	90	86	37	277	46	39	0.8	0.5	0.3	0.7	115.4	20	6	3.9
RL3	41	62	1.56	25.5	130	70	99	100	37	250	44	42	0.5	0.3	0.2	0.8	75.9	24	5.7	2.9
RL4	55	55	1.36	29.8	110	70	97	84	37	294	54	49	0.9	0.5	0.4	0.9	66.3	27	5.9	3.5
RL5	42	67	1.36	36.4	130	70	99	93	37	302	58	56	1.1	0.8	0.3	0.7	88.6	55	5.7	2.7
RL6	64	85	1.46	39.9	120	80	94	143	37	297	50	53	0.9	0.6	0.3	0.8	102.7	19	5.6	3.8
RL7	48	55	1.55	22.9	150	110	97	74	37	288	60	55	0.6	0.6	0.2	1.1	68	29	6.5	3.4
RL8	42	72	1.54	30.3	140	80	99	84	37	261	50	46	0.8	0.5	0.3	1.8	38.7	33	6.3	2.9
RL9	35	54	1.5	24	140	80	99	84	37	289	54	49	1	0.4	0.6	0.8	100	19	5.9	1.9
RL10	55	55	1.63	20.7	120	80	99	91	37	240	35	33	0.6	0.3	0.3	2.6	18.5	81	5.9	2.7
RL11	32	58	1.53	24.7	120	80	95	67	37	299	49	46	1	0.6	0.4	0.6	103	21	5.7	2
RL12	29	63	1.53	26.9	120	80	99	82	37	249	38	36	0.8	0.6	0.2	2.5	19.4	59	5.8	2.8
RL13	55	50	1.52	21.6	120	80	96	141	37	280	46	44	0.9	0.6	0.3	0.9	66.5	17	5.6	2.5
RL14	54	51	1.5	22.6	140	90	95	143	37	973	36	38	0.5	0.3	0.2	0.7	87.2	16	5.3	3.5
RL15	45	64	1.46	30	170	90	95	102	37	212	23	28	0.4	0.2	0.2	0.8	72.9	16	5.7	3
RL16	45	63	1.5	28	120	70	95	91	37	430	15	28	0.8	0.2	0.6	0.6	103	19	8.1	1.4
RL17	42	71	1.52	30.7	180	90	97	93	37	358	29	20	0.8	0.2	0.6	0.8	74.7	14	7.5	3.4
RL18	43	50	1.5	22.2	110	70	97	84	37	294	54	49	0.8	0.5	0.3	0.6	104.2	16	3.2	2.9
RL19	55	55	1.36	29.8	140	80	99	84	37	288	60	55	0.9	0.3	0.6	0.7	90.5	29	6.9	2.7
RL20	53	54	1.5	24	120	80	99	91	37	240	35	33	0.6	0.3	0.3	0.6	110.7	20	6.1	3.5
RL21	45	50	1.5	22.2	120	80	94	100	37	289	50	52	0.9	0.5	0.4	0.8	100	26	5.9	2.9
RL22	42	60	1.5	26.6	120	80	90	86	37	277	46	39	0.8	0.5	0.3	0.7	115.4	20	6	3.9
RL23	41	62	1.56	25.5	130	70	99	100	37	250	44	42	0.5	0.3	0.2	0.8	75.9	24	5.7	2.9
RL24	49	55	1.36	29.8	110	70	97	84	37	294	54	49	0.9	0.5	0.4	0.9	66.3	27	5.9	3.5
RL25	46	67	1.36	36.4	130	70	99	93	37	302	58	56	1.1	0.8	0.3	0.7	88.6	55	5.7	2.7

RL26	38	85	1.46	39.9	120	80	94	143	37	297	50	53	0.9	0.6	0.3	0.8	102.7	19	5.6	3.8
RL27	49	55	1.55	22.9	150	110	97	74	37	288	60	55	0.6	0.6	0.2	1.1	68	29	6.5	3.4
RL28	37	72	1.54	29.5	140	80	99	84	37	261	50	46	0.8	0.5	0.3	1.8	38.7	33	6.3	2.9
RL29	41	54	1.5	24	140	80	99	84	37	289	54	49	1	0.4	0.6	0.8	100	19	5.9	1.9
RL30	42	55	1.63	20.7	120	80	99	91	37	240	35	33	0.6	0.3	0.3	2.6	18.5	81	5.9	2.7
RL31	53	58	1.53	24.7	120	80	95	67	37	299	49	46	1	0.6	0.4	0.6	103	21	5.7	2
RL32	61	63	1.53	26.9	120	80	99	82	37	249	38	36	0.8	0.6	0.2	2.5	19.4	59	5.8	2.8
RL33	52	50	1.52	21.6	120	80	96	141	37	280	46	44	0.9	0.6	0.3	0.9	66.5	17	5.6	2.5
RL34	47	51	1.5	22.6	140	90	95	143	37	973	36	38	0.5	0.3	0.2	0.7	87.2	16	5.3	3.5
RL35	47	64	1.46	30	170	90	95	102	37	212	23	28	0.4	0.2	0.2	0.8	72.9	16	5.7	3
RL36	50	63	1.5	28	120	70	95	91	37	430	15	28	0.8	0.2	0.6	0.6	103	19	8.1	1.4
RL37	48	71	1.52	30.7	180	90	97	93	37	358	29	20	0.8	0.2	0.6	0.8	74.7	14	7.5	3.4
RL38	42	50	1.5	22.2	110	70	97	84	37	294	54	49	0.8	0.5	0.3	0.6	104.2	16	3.2	2.9
RL39	39	55	1.36	29.8	140	80	99	84	37	288	60	55	0.9	0.3	0.6	0.7	90.5	29	6.9	2.7
RL40	35	54	1.5	24	120	80	99	91	37	240	35	33	0.6	0.3	0.3	0.6	110.7	20	6.1	3.5
RL41	54	50	1.5	22.2	120	80	94	100	37	289	50	52	0.9	0.5	0.4	0.8	100	26	5.9	2.9
RL42	62	60	1.5	26.6	120	80	90	86	37	277	46	39	0.8	0.5	0.3	0.7	115.4	20	6	3.9
RL43	43	62	1.56	25.5	130	70	99	100	37	250	44	42	0.5	0.3	0.2	0.8	75.9	24	5.7	2.9
RL44	45	55	1.36	29.8	110	70	97	84	37	294	54	49	0.9	0.5	0.4	0.9	66.3	27	5.9	3.5
RL45	48	67	1.36	36.4	130	70	99	93	37	302	58	56	1.1	0.8	0.3	0.7	88.6	55	5.7	2.7
RL46	41	85	1.46	39.9	120	80	94	143	37	297	50	53	0.9	0.6	0.3	0.8	102.7	19	5.6	3.8
RL47	42	55	1.55	22.9	150	110	97	74	37	288	60	55	0.6	0.6	0.2	1.1	68	29	6.5	3.4
RL48	46	72	1.54	30.3	140	80	99	84	37	261	50	46	0.8	0.5	0.3	1.8	38.7	33	6.3	2.9
RL49	44	54	1.5	24	140	80	99	84	37	289	54	49	1	0.4	0.6	0.8	100	19	5.9	1.9
RL50	49	55	1.63	20.7	120	80	99	91	37	240	35	33	0.6	0.3	0.3	2.6	18.5	81	5.9	2.7
