# Supplementary material for: Comparative analysis of multiorgan toxicity induced by long term use of disease modifying anti-rheumatic drugs
Source: PLoS One. 2023 Aug 25;18(8):e0290668. doi: 10.1371/journal.pone.0290668 (PMC10456141; doi:10.1371/journal.pone.0290668)
Supplement: Attachment — Submitted filename: Review Report PONE-D-23-10597.docx [file pone.0290668.s006.docx]

**Review Report:** Paper Number: PONE-D-23-10597….

**Tittle of the manuscript: PLOS ONE Comparative Analysis of Multiorgan Toxicity Induced by Long Term Use of Disease Modifying Anti-Rheumatic Drugs**

Dear Editor,

The article " Comparative Analysis of Multiorgan Toxicity Induced by Long Term Use of Disease

Modifying Anti-Rheumatic Drugs" was reviewed. It is a good study. The authors describe the potential of both drugs prescribed as treatment of arthritis. I recommend the publication of manuscript after some revision.

The authors have done the good research work and their work is appreciable. But I find some of the points according to my knowledge.

I have appended below some comments that the authors may wish to consider:

1. Page 2: Abstract: Under Methodology ‘‘Stress on liver was estimated by measuring change in liver enzymes (ALT, AST, and ALP) and total bilirubin. While, degree of renal impairment was assessed by calculating GFR’’ the abbreviations are not preferred in the abstract so author should be mentioned the complete names.
2. Page 3: Introduction: In the last paragraph ‘’ First, females have stronger and more reactive immune system than men, secondly, feminine hormones also flare RA risks’’. These reasons not clear the author claim in the previous lines. It should be clearer.
3. Page 5: Materials and methods: the author has claimed the teen agers not included in the study. It is better to mention the age limit also.
4. Page 7: Result: same reservation about age. It is mentioned middle age and last decade of age which is not clear the proper age limit.
5. Page 8: it is stated that ‘‘LEF treatment also speeds up the pulse rate (10.8%) and this elevation is statistically significant (P=0.0002)’’. It may be corrected as (p<0.0002). It should be properly checked according to the value mentioned in the table.
6. Page 13: Discussion section 2^nd^ paragraph. The author discusses about the problem with the age 53 included in the study where as they have not mentioned clearly the age limits in the materials, so more explanation needed here
7. References: All the references mentioned in the references list are not according to the instructions provided in the author guidelines.
8. The overall format of the manuscript needs revision according to the author guide line provided by the Plose One: abstract should be in paragraph form. Format of the headings also needs revision. Reference style also needs revision: full Journal names are mentioned in the references it should be abbreviated.
9. All figures are difficult to read or understand due to pixel problem might be. They should be properly insert according to the guidelines.
