# Supplementary material for: Comparative analysis of multiorgan toxicity induced by long term use of disease modifying anti-rheumatic drugs
Source: PLoS One. 2023 Aug 25;18(8):e0290668. doi: 10.1371/journal.pone.0290668 (PMC10456141; doi:10.1371/journal.pone.0290668)
Supplement: Attachment — Submitted filename: Response to reviewers.docx [file pone.0290668.s007.docx]

**Dear Dr. Samiullah Khan**

**Academic Editor**

**PLOS ONE**

All the authors are very thankful for considering the manuscript for peer review. We are much obliged for valuable comments and suggestions of academic editor and reviewers to improve the quality of the article. The manuscript has been revised accordingly. A detailed, point to point response to the academic editor and reviewers’ comments is as follow

**Response to Academic Editor**

1. There is no change in financial disclosure statement. This research work did not receive any grant from funding agency in the public, commercial or industrial sector.
2. The protocols used in the study are recommended by IFCC (International Federation of Clinical Chemistry and Laboratory Medicine) through commercially available kits developed by DiaSys Diagnostic Systems GmbH Germany. The made and catalogue numbers of all kits are mentioned in revised manuscript. We are agreed to your recommendation of submitting laboratory protocols in protocols.io to enhance the reproducibility of results. We will definitely work on it.

**Response to Journal Requirements**

**PLOS ONE stye requirements**

1. The manuscript has been revised according to PLOS ONE formatting guidelines for main body of manuscript, authors affiliations and file naming. The authors have tried our level best to make the article as per journal style requirement but still man is prone to error, even if a mistake is made, we are ready to correct it.
2. The manuscript has been thoroughly checked and proof read for language use, spelling and grammar by the authors themselves and no professional services for copyedit is used.

A copy of manuscript showing changes using “track changes” has been uploaded as a *supporting information* file. Revised version of manuscript without track changes named as “manuscript” has been uploaded

3. We apologize for misunderstanding the purpose of data availability statement. All the study’s minimal data sets with anonymous patient ID have been uploaded as supporting information files.
4. We feel sorry for the poor quality of figures and are much thankful to have PACE – the Picture Analysis and Conversion Engine (<https://pacev2.apexcovantage.com>). The application has made it very easy to convert image into required format. In fact, I learned a lot while revising the manuscript for PLOS ONE

**Response to reviewer 1(Khola Noureen)**

The authors are much obliged for such an in-depth review. In deed it was a very comprehensive review and all aspect of the study were observed with eagle eye. This detailed review helped us a lot to make the article worth writing. Following are satisfactory answers of the comments

1. **Title was “Comparative Analysis of Multiorgan Toxicity Induced by Long Term Use of Disease Modifying Anti-Rheumatic Drugs”, ideally title should Indicate the study’s design with a commonly used term in the title or the abstract**

As for as title is concerned, it has no space for study design. Generally, study design is defined in abstract and is mentioned in abstract (page 2, line 3-4)

2. **Study objective is not properly specified, state specific objectives, including any pre-specified hypotheses**.

In deed it is a valid point. Study objectives are thoroughly considered and revised as per suggestion. Specifically. Underlying hypothesis and specific objectives are included in Abstract “objective section” (page 2 line 2-5)

3. **The title, objective and rationale of the study are not aligned.**

The title, objective and rationale of study are revised and integrated to each other (page 4 line 59-66)

4. **Number of controls was half as compared to a number of cases**

The ratio of case to control is1:1. Actually the number of participants in each group was equal. The control subjects were same for each case group (Fifty healthy subjects were recruited against fifty (50) RA patients treated with MTX and the same healthy subjects were compared against RA patients treated with LEF. There were two basic reasons for same control group

a. The healthy individuals were fully screened for complete medical and physical fitness and subsequently found to be perfectly healthy subjects
b. Since the study was not funded by anywhere, we compared the same control for both groups considering the financial burden.
5. **Control must be matched for certain variables like sex and age**

Revised as suggested (page 5 Line 87-88)

6. **The eligibility criteria, and the sources and methods of case ascertainment and control selection is not clear, how matching of cases and controls was addressed any sensitivity analyses. It is better to report numbers of individuals at each stage of study e.g. numbers potentially eligible, examined for eligibility, confirmed eligible, included in the study, completing follow-up, and analyzed, reasons for non-participation at each stage, consider use of a flow diagram to make it clear and explicit.**

Revised. See Supporting Information S1 Fig (page 5 line 81)

7. **There is no explanation about how the study size was arrived**

Explained on Page 5 line 86-87

8. **Data handling of quantitative variables is not explained properly. Statistical analysis lacks an explanation of how confounding subgroups and interactions are handled; how missing data were addressed. In confounder-adjusted estimates and their precision (eg, 95% confidence interval) is not explained**.

STROBE check list is attached as other supporting document

9. **Percentage variation is not done for control group, justification for using Mean, SEM and significance of statistical difference is not clear**

Mean values of each case group were compared with mean value of control group to calculate the percentage difference from control group. As mean value of control group was used as reference, percentage variation cannot be done for standard or reference value.

10. **Conclusion do not support overall interpretation of results considering objectives, limitations, results from similar studies, and other relevant evidence.**

Conclusion has been revised in light of objectives, study limitations and results (page 16, line 295-304).

**Response to Reviewer 2**

The authors are very much grateful for your positive reviews. Your positive approach and encouragement have shown us a new path. We are happy to implement your valuable suggestion and feel pleasure to answers your kind questions. The points are as follows

1. **Page 2: Abstract: Under Methodology ‘‘Stress on liver was estimated by measuring**

**change in liver enzymes (ALT, AST, and ALP) and total bilirubin. While, degree of renal**

**impairment was assessed by calculating GFR’’ the abbreviations are not preferred in the**

**abstract so author should be mentioned the complete names.**

Incorporated as suggested (page 2 line 12-14)

2. **Page 3: Introduction: In the last paragraph ‘’ First, females have stronger and more reactive immune system than men, secondly, feminine hormones also flare RA risks’’. These reasons not clear the author claim in the previous lines. It should be clearer.**

Statement made more clearer (page 3 line 43-45)

3. **Page 5: Materials and methods: the author has claimed the teen agers not included in the study. It is better to mention the age limit also.**

Age was already mentioned on page 5 line 96. However, for more convenient reading, the word teenager replaced with “participants of less than 18 years” (Page 5 line 84)

4. **Page 7: Result: same reservation about age. It is mentioned middle age and last decade of age which is not clear the proper age limit.**

Age range has been mentioned (Page 7 line 129-131)

5. **Page 8: it is stated that ‘‘LEF treatment also speeds up the pulse rate (10.8%) and this elevation is statistically significant (P=0.0002)’’. It may be corrected as (p<0.0002). It should be properly checked according to the value mentioned in the table.**

Very good observation. Authors appreciated the keen interest of reviewer in result interpretation. It was really a mistake. Results has been carefully checked and revised accordingly (page 8 line 158, page 9 Table 1)

6. **Page 13: Discussion section 2nd paragraph. The author discusses about the problem with the age 53 included in the study where as they have not mentioned clearly the age limits in the materials, so more explanation needed here**

The average age of study participant was 45 years. It has been mentioned in Results section under heading “age distribution” on page 7 line 130. Correction in average age has been made on page 15 line 283.

7. **References: All the references mentioned in the references list are not according to the instructions provided in the author guidelines.**

References are formatted according to guideline.

8. **The overall format of the manuscript needs revision according to the author guide line provided by the PLOS One: abstract should be in paragraph form. Format of the headings also needs revision. Reference style also needs revision: full Journal names are mentioned in the references it should be abbreviated.**

The whole manuscript has been revised and prepared according to PLOS ONE guidelines

**We believe that manuscript is now suitable for publication in PLOS ONE**

Best Regard

Dr. Tamseela Mumtaz (On behalf of all authors)

Assistant Professor, Department of Zoology

Government College Women University, Faisalabad
