# Supplementary material for: Comparative analysis of multiorgan toxicity induced by long term use of disease modifying anti-rheumatic drugs
Source: PLoS One. 2023 Aug 25;18(8):e0290668. doi: 10.1371/journal.pone.0290668 (PMC10456141; doi:10.1371/journal.pone.0290668)
Supplement: Attachment — Submitted filename: Reviewer coments RA paper agaIN 2023.docx [file pone.0290668.s008.docx]

**Review Report:** Paper Number: PONE-D-23-10597….

**Tittle of the manuscript: PLOS ONE Comparative Analysis of Multiorgan Toxicity Induced by Long Term Use of Disease Modifying Anti-Rheumatic Drugs**

Dear Editor,

Dear Editor,

The article " Comparative Analysis of Multiorgan Toxicity Induced by Long Term Use of Disease

Modifying Anti-Rheumatic Drugs" was again reviewed after the corrections made by author.

I have read the manuscript in details and observed that authors have made corrections in all the aspects I asked previously. I find it now properly described everything for the publication. I think the author meet all the criteria of journal provided guide lines.

The editor may accept this paper for the publication in the Plose One Journal.
