# Supplementary material for: Comparative analysis of multiorgan toxicity induced by long term use of disease modifying anti-rheumatic drugs
Source: PLoS One. 2023 Aug 25;18(8):e0290668. doi: 10.1371/journal.pone.0290668 (PMC10456141; doi:10.1371/journal.pone.0290668)
Supplement: Attachment — Submitted filename: Response to Reviewers.docx [file pone.0290668.s009.docx]

**Dear Dr. Samiullah Khan**

**Academic Editor**

**PLOS ONE**

My co-authors and I were pleased to receive your response on 5^th^ July 2023 inviting us for minor revision and resubmission of article entitled “Comparative analysis of multiorgan toxicity induced by long term use of disease modifying anti-rheumatic drugs” (manuscript ID PONE-D-23-10597R1). We are very much obliged for your precious time and efforts in reviewing our manuscript. Your quick comments are very helpful in improving the contents and presentations of our manuscript. Accordingly, we added the recent references to make the article up to date. The point wise answers of the journal requirement and editor comments are as under,

| **Sr #** | **Journal requirement** | **Response** |
| --- | --- | --- |
| 1 | Please review your reference list to ensure that it is complete and correct. If you have cited papers that have been retracted, please include the rationale for doing so in the manuscript text, or remove these references and replace them with relevant current references. | It is made sure that reference list is complete and correct. No retracted paper has been cited. All the quoted references are correct and valid. |

| **Sr.#** | **Editor comments** | **Response** |
| --- | --- | --- |
| 1 | The author should also include some recent relevant (2021-2023) references in the introduction as well as in the discussion section | Recent references have been added into introduction and discussion section (See Ref 1, line # 38, Ref 9, line # 56, Ref 19, line # 261-263, Ref 21 line # 269 |

On behalf of all my co-authors, I would like to extend my gratitude for positive and constructive remarks of both reviewers. The comments of reviewer 1 are valid and depicted the reviewer strong epidemiological/statistical background. The points raised on sample size and sample recruitment/selection are very logical and draw our attention to a very important point which we forgot to address. The recommendations and advices help us a lot in improving the quality of the article. We have revised the manuscript according to the reviewer comments and explained all the points under “response”. The tracked manuscript also showed minor revision and added/ renumbered reference. The point-to-point response of the reviewer comments are as under

|  | **Reviewer 1 comments** | **Response** |
| --- | --- | --- |
| 1 | Reviewer wrote all of the abstract under heading of objective, statement starting from for this purpose ....is part of methodology rather than objective... | Heading “objective” was a typographical error. It has been resolved. |
| 2 | .... Abstract is unstructured, all has been written under one heading | As per submission guidelines, the abstract should not be divided into headings and must be in one paragraph describing objectives, methodology, main results, and conclusion. So abstract is in one paragraph according to guidelines |
| 3 | (Number of participants allocated to each group must be mentioned. There should be a clear description as how 100 cases and 50 controls were allocated into different group specially mentioning the number of each group , since its a case control study , this information is very pertinent as ratio of allocation of cases to control and for matched studies, its important to give matching criteria and the number of controls per case which is not clear even in revised manuscript . | Done (see Abstract section line # 8, Materials and methods section, subsection sample population, line # 88-94) |
| 4 | In line 94, 95 its mentioned "chosen rheumatoid arthritis subjects were categorized into two subgroups based on oral administration of two different types of DMARDs (Group 1: methotrexate (n=50), Group 2: leflunomide (n=50) "however this statement has no mention of allocation of controls into each group | Done (Materials and method section, subsection Sample population line # 94) Supporting information S1 file) |
| 5 | Sample size estimation is still not done as per statistical rule, sample size calculation in statistical terms is based on determination of the required number of cases and controls is based on consideration of the strength of the relationship between the disease and exposure to the putative cause, the variability in exposure within the population under study, and the desired size and power of the statistical test, which is still missing. | Sample size estimation was done by G* Power software. Details are incorporated in Materials and methods section, sub section Study design line # 73-75 |
| 6 | Results: under heading of result ..line 127 , its mentioned "Overall, 80% patients (n=80) were treated with comparatively low dose (10 mg)" author should mention the potential confounding effect of variation in doses | Done (line 135-136) |
| 7 | side effects mentioned "Both methotrexate and leflunomide had some side effects such as fatigue, headache, alopecia, teeth pain, rash, dizziness, sleep problem, cough, depression, breathing problem and muscle stretching" are quite vague, author should operationally define these terms and explicitly mention how these complaints were measured | These side effects have been reported in number of researches thus supported by literature review. (Fraenkel et al., 2004; Wanying et al., 2018) As for as this study is concerned, a comprehensive questionnaire was design to collect all the information regarding side effects. (S1 file) These are the generalized symptoms/terms that patients reported while using the prescribed drug. However, different scales were consulted for reporting the severity of adverse effects of the treatment such as NPRS (numeric pain rating scale) for headache, visual analogue scale for pain intensity, fatigue assessment scale for fatigue, Ludwig scale for hair loss and Sleep quality scale (SQS) for insomnia. As the number of samples reporting adverse effects were limited, the severity could not be categorized (mild, moderate, severe etc) These variables have no effect on study outcomes (see line #146-147)  Supporting Reading  Wanying Wang, Hua Zhou, Liang Liu, (2018) Side effects of methotrexate therapy for rheumatoid arthritis: A systematic review, European Journal of Medicinal Chemistry, Volume 158, Pages 502-516, ISSN 0223-5234, <https://doi.org/10.1016/j.ejmech.2018.09.027>.  Fraenkel L, Bogardus ST, Concato J, et al Patient preferences for treatment of rheumatoid arthritis Annals of the Rheumatic Diseases 2004;63:1372-1378. |
| 8 | in line 174 and 175 " patients have high heart rates that may be attributed to other co morbidities like obesity, insulin resistance etc" these are potential confounders which need to be control during participants recruitment phase | This sentence was written mistakenly and all these patients were excluded while recruiting the participant by implementing the exclusion criteria. Thus, all such potential confounders were controlled at the time of sample selection. Therefor this line has been removed |

After careful response to every point, we would like to submit the revised manuscript for reconsideration for publication in PLOS One Journal.

We also thanks to reviewer 2 for recommended the article accepted for publication

**We believe that manuscript is now suitable for publication in PLOS ONE**

Best Regard

Dr. Tamseela Mumtaz (On behalf of all authors)

Assistant Professor, Department of Zoology

Government College Women University, Faisalabad
